# Draft Genome Sequence of *Erwinia toletana*, a Bacterium Associated with Olive Knots Caused by *Pseudomonas savastanoi* pv. Savastanoi

**DOI:** 10.1128/genomeA.00205-13

**Published:** 2013-05-09

**Authors:** Daniel Passos da Silva, Giulia Devescovi, Konrad Paszkiewicz, Chiaraluce Moretti, Roberto Buonaurio, David J. Studholme, Vittorio Venturi

**Affiliations:** International Centre for Genetic Engineering and Biotechnology, Trieste, Italya;; Biosciences, University of Exeter, Exeter, Devon, United Kingdomb;; Dipartimento di Scienze Agrarie e Ambientali, Università degli Studi di Perugia, Perugia, Italyc

## Abstract

*Erwinia toletana* was first reported in 2004 as a bacterial species isolated from olive knots caused by the plant bacterium *Pseudomonas savastanoi* pv. savastanoi. Recent studies have shown that the presence of this bacterium in the olive knot environment increases the virulence of the disease, indicating possible interspecies interactions with *P. savastanoi* pv. savastanoi. Here, we report the first draft genome sequence of an *E. toletana* strain.

## GENOME ANNOUNCEMENT

*Erwinia toletana* is a nonpathogenic Gram-negative member of the *Enterobacteriaceae* family, and it was isolated in 2004 from knots induced by *Pseudomonas savastanoi* pv. savastanoi in olive plants (*Olea europaea* L.) (1). It is currently unclear whether *E. toletana* in the olive knots is an epiphyte, an endophyte, or both. Recent studies have shown that olive plants inoculated with both the harmless *E. toletana* and the pathogen *P. savastanoi* pv. savastanoi develop more severe disease than plants inoculated with *P. savastanoi* pv. savastanoi alone. One of the possible mechanisms proposed for this effect is sharing, between the two species, of *N-*acyl homoserine lactone quorum-sensing signals and the formation of a stable interspecies community (2).

Here, we announce the draft genome sequence of *E. toletana* strain DAPP-PG 735, isolated in Italy from an olive knot caused by *P. savastanoi* pv. savastanoi (2). The genome sequence was determined using a 36-bp paired-end library with the Illumina GA sequencing system, as described previously (3). We obtained a total of 33,027,878 pairs of reads, representing approximately 200-fold coverage of the genome. We performed *de novo* assembly using Velvet 1.1.03 (4), generating 94 contigs that were further joined into 23 scaffolds with a mean length of 226 kbp. The total length of the scaffold assembly is 5.2 Mbp, and the *N*_50_ length is 576 kbp, assuming a genome size of 5.3 Mb. The longest scaffold obtained is 2.2 Mbp. The G+C content is 53.6%, which is consistent with that observed by Rojas et al. (1) and which is similar to those of other sequenced *Erwinia* genomes. Automated annotation of the *E. toletana* draft genome sequence using RAST (5) assigned a total of 3,709 candidate protein-coding genes. Among all the predicted genes, a total of 1,057 genes were annotated as encoding hypothetical proteins. A total of 3 rRNA and 49 tRNA genes were also identified in the RAST annotation. Among several interesting features uncovered by the automatic annotation of the *E. toletana* genome is the presence of a large number of genes involved in the utilization of aromatic compounds as a carbon source. This ability to transform a large set of plant-related aromatic compounds for detoxification or metabolic purposes might be significant, since plants are also known to produce them as defense mechanisms against microorganisms (6, 7). This report presents the first draft genome sequence of an *E. toletana* strain.

### Nucleotide sequence accession numbers.

This Whole-Genome Shotgun project has been deposited at DDBJ/EMBL/GenBank under the accession no. AOCZ00000000. The version described in this paper is the first version, accession no. AOCZ01000000.
